# Association of the Chronotype Score with Circulating Trimethylamine N-Oxide (TMAO) Concentrations

**DOI:** 10.3390/nu13051671

**Published:** 2021-05-14

**Authors:** Luigi Barrea, Giovanna Muscogiuri, Gabriella Pugliese, Chiara Graziadio, Maria Maisto, Francesca Pivari, Andrea Falco, Gian Carlo Tenore, Annamaria Colao, Silvia Savastano

**Affiliations:** 1 Dipartimento di Scienze Umanistiche, Università Telematica Pegaso, 80143 Napoli, Italy; 2 Centro Italiano per la cura e il Benessere del Paziente con Obesità (C.I.B.O), Endocrinology Unit, Department of Clinical Medicine and Surgery, University Federico II, 80131 Naples, Italy; giovanna.muscogiuri@gmail.com (G.M.); robiniapugliese@gmail.com (G.P.); chiaragraziadio@live.it (C.G.); operafederico2@gmail.com (A.C.); sisavast@unina.it (S.S.); 3 Unit of Endocrinology, Dipartimento di Medicina Clinica e Chirurgia, Federico II University Medical School of Naples, 80131 Naples, Italy; 4 Cattedra Unesco “Educazione alla Salute e allo Sviluppo Sostenibile”, University Federico II, 80131 Naples, Italy; 5 Department of Pharmacy, University of Naples “Federico II”, 80131 Naples, Italy; maria.maisto@unina.it (M.M.); giancarlo.tenore@unina.it (G.C.T.); 6 Department of Health Sciences, University of Milan, 20142 Milan, Italy; francesca.pivari@unimi.it; 7 Department of Science and Technology (DST), Università del Sannio, 82100 Benevento, Italy; falco.and@gmail.com

**Keywords:** chronotype, trimethylamine N-oxide (TMAO), Mediterranean diet, diet, nutritionist

## Abstract

Individual differences in the chronotype, an attitude that best expresses the individual circadian preference in behavioral and biological rhythms, have been associated with cardiometabolic risk and gut dysbiosis. Up to now, there are no studies evaluating the association between chronotypes and circulating TMAO concentrations, a predictor of cardiometabolic risk and a useful marker of gut dysbiosis. In this study population (147 females and 100 males), subjects with the morning chronotype had the lowest BMI and waist circumference (*p* < 0.001), and a better metabolic profile compared to the other chronotypes. In addition, the morning chronotype had the highest adherence to the Mediterranean diet (*p* < 0.001) and the lowest circulating TMAO concentrations (*p* < 0.001). After adjusting for BMI and adherence to the Mediterranean diet, the correlation between circulating TMAO concentrations and chronotype score was still kept (r = −0.627, *p* < 0.001). Using a linear regression analysis, higher chronotype scores were mostly associated with lower circulating TMAO concentrations (β = −0.479, *t* = −12.08, and *p* < 0.001). Using a restricted cubic spline analysis, we found that a chronotype score ≥59 (*p* < 0.001, R^2^ = −0.824) demonstrated a more significant inverse linear relationship with circulating TMAO concentrations compared with knots <59 (neither chronotype) and <41 (evening chronotype). The current study reported the first evidence that higher circulating TMAO concentrations were associated with the evening chronotype that, in turn, is usually linked to an unhealthy lifestyle mostly characterized by low adherence to the MD.

## 1. Introduction

Circadian rhythms, endogenously driven cycles entrained to the 24 h external environment, exert broad-ranging control over many biological processes, including feeding behavior, the activity of the gastrointestinal system, and gut microbiota [1]. On the other side, a plausible role for the feeding behavior and gut microbiota in modifying metabolism and circadian rhythms has been well described [2]. In humans, there are large interindividual differences in behavioral and biological rhythms [3,4], which lead to different circadian phenotypes, named chronotypes [5]. In particular, there are three different chronotypes expressed as a continuum between two extremes, from morningness to eveningness, with a third in an intermediate position. The morning chronotype is associated with greater morning energy to go to bed earlier and wake up early, and, in summary, a preference for diurnal activities [6]. Contrariwise, the evening chronotype is related to irregular sleep and wake-up patterns, later sleep and rising, with a tendency for nocturnal activities [7,8,9]. Disruption of the endogenous central and peripheral clocks is associated with adverse health outcomes, such as excess weight, metabolic dysfunctions, and gut dysbiosis [10]. Gut dysbiosis is an abnormal intestinal microbiota community of organisms resident in the gastrointestinal tract, with increased host susceptibility to pro-inflammatory diseases [11]. Several epidemiological studies have shown that the evening chronotype tends to have more health problems and greater mortality compared to the morning chronotype [12,13]. More recently, a dangerous connection has been established between a blunted microbial profile and unhealthy feeding behavior in subjects with the evening chronotype [14].

Trimethylamine (TMA) is the product of the metabolism of dietary nutrients commonly present in the Western diet, such as choline and carnitine, which are found in red meat, eggs, milk, and cheese [15]. TMA is further metabolized in the liver to trimethylamine N-oxide (TMAO) [15]. Despite the fact that there is a large consensus that circulating TMAO concentrations are mainly dependent on dietary patterns, a relevant contribution to the liver production of TMAO is provided by the fermentation of dietary TMA produced by gut bacteria, including *Proteobacteria* and *Firmicutes* [16]. Through mediating inflammatory processes, TMAO would have a potential association with most chronic non-communicable diseases, including cancer, type 2 diabetes, obesity, metabolic syndrome, nonalcoholic fatty liver disease, and Alzheimer’s disease [15]. In the case of gut dysbiosis, an increase in the microorganisms that produce TMAO has been observed, thus suggesting TMAO as one of the biomarkers for metabolite profiling and diagnostic suitability for dysbiosis [17].

The Mediterranean dietary pattern, along with physical activity promotion and circadian clock entrainment, seems to represent a useful combination for dealing with several chronic non-communicable diseases [18]. In addition, the Mediterranean dietary pattern is also the most characterized factor that influences the gut microbiota [19]. The complexity of studying the gut microbiota is, however, characterized by a high intra-individual variability of the daily food consumption of each subject that might potentially hinder the robustness and reproducibility of microbiota—disease associative studies [20]. We previously reported that the lowest adherence to the Mediterranean diet (MD) was associated with the highest circulating TMAO concentrations [21]. Up to now, there are no studies evaluating the association between the three chronotypes and circulating TMAO concentrations.

Considering the influence of the MD on both chronotypes and circulating TMAO concentrations, this study aimed to evaluate the possible association of the three chronotypes with circulating TMAO concentrations in a sample of the adult population according to sex, lifestyle habits, BMI, and adherence to the MD.

## 2. Materials and Methods

### 2.1. Design and Setting

We carried out an observational cross-sectional study from December 2016 to January 2021 at the Department of Clinical Medicine and Surgery, Unit of Endocrinology, University Federico II, Naples (Italy). The Ethical Committee “Federico II” approved this study (*n*. 173/16), which was carried out following the Code of Ethics of the World Medical Association (Declaration of Helsinki) for clinical studies. All study participants, after being informed of the purpose and procedures, provided informed consent.

### 2.2. Population Study

Two hundred forty-seven Caucasian adult subjects aged between 20 and 68 years of both sexes were consecutively enrolled in the same urban area in Naples in Italy among patients of our clinic, volunteers, hospital employees, and participants in the Obesity, Programs of nutrition, Education, Research and Assessment of the best treatment (OPERA) project [22]. It was confirmed that all women were neither pregnant nor lactating. The pathological and pharmacological anamnesis of all participating subjects was collected. We decided to enroll only adults aiming at increasing sample homogeneity, and the following exclusion criteria were applied:

- Impaired renal function (estimated glomerular filtration rate calculated by chronic kidney disease epidemiology collaboration equation <90 mL/min/1.73 m^2^);
- A confirmed diagnosis of type 2 diabetes (in accordance with the American Diabetes Association criteria: two confirmations of fasting glucose ≥126 mg/dL or glycated hemoglobin (HbA1c) ≥6.5% (≥48 mmol/mol)). Subjects taking hypoglycemic drugs were considered to have type 2 diabetes [23];
- Cardiovascular disease, including: previous cardiovascular events, atherosclerosis, coronary artery or peripheral vascular disease;
- Current use of hypolipidemic or anti-inflammatory drugs;
- Current therapy or in the two months prior to enrollment with antibiotics or probiotics;
- Specific diets such as vegetarian or vegan regimes;
- Current supplementation with vitamins, minerals, or antioxidants;
- Alcohol abuse was diagnosed according to the criteria of DSM-V [24];
- Shift working.

### 2.3. Lifestyle Information

Information on lifestyle, including cigarette smoking habits and physical activity, was collected from all participants. In particular, current smokers have been defined as those who smoke at least one cigarette a day, former smokers are those who have not smoked for at least a year, while all others have been defined as non-current smokers. If subjects performed at least 30 min of physical activity per day, they were classified as “Physical activity YES”, if not “Physical activity NO”, as previously reported in other studies [25,26,27]. 

### 2.4. Anthropometric Measurements and Blood Pressure

All participants were evaluated in the morning after at least 8 h of fasting by the same nutritionist with proven experience in assessing body composition and nutritional status, as previously reported [28,29,30]. All subjects were measured wearing light clothing and without shoes: height was obtained using a wall-mounted stadiometer (Seca 711; Seca, Hamburg, Germany) and body weight was assessed with a calibrated balance beam scale (Seca 711; Seca, Hamburg, Germany) to the nearest 0.1 kg. Weight and height were measured to calculate the body mass index (BMI) (weight (kg) divided by height squared (m^2^), kg/m^2^), which was used to classify subjects in accordance with WHO’s criteria as normal weight: 18.5–24.9 kg/m^2^; overweight, 25.0–29.9 kg/m^2^; grade I obesity, 30.0–34.9 kg/m^2^; grade II obesity, 35.0–39.9 kg/m^2^; grade III obesity ≥40.0 kg/m^2^ [31]. Waist circumference (WC) was obtained using a non-stretchable measuring tape to the closest 0.1 cm according to the National Center for Health Statistics [32], at the narrowest point of the abdomen or, if this was not visible, at the point between the lower edge of the rib cage and the iliac crest. Systolic and diastolic blood pressure (SBP and DBP, respectively) were measured in all subjects three times using a random zero sphygmomanometer (Gelman Hawksley Ltd., Sussex, UK) after 10 min sitting at rest, finally reporting the average of the last two values. 

### 2.5. Determination of Circulating TMAO Concentrations

Circulating TMAO concentrations were obtained in all participants and samples were stored at −80 °C, having been shown that under these conditions TMAO remains stable for years [33]. Circulating TMAO concentrations were measured using the method described by Beale and Airs [34], and reported in our previous study [35,36,37]. The chromatographic separation was carried out with a guard column (HILIC), in combination with a Luna HILIC column (150 × 3 mm, 5 µm particles), both supplied by Phenomenex (Torrance, CA, USA).

### 2.6. Assay Methods

All participants underwent a blood draw during the morning (8–10 a.m.) in a state of fasting for at least 8 h and all samples were stored at −80 °C until analysis. Roche Modular Analytics System was used to perform all biochemical analyses including fasting glucose, lipid profile, and transaminases, and a direct method of homogeneous enzymatic assay was used to measure low-density lipoprotein (LDL) cholesterol and high-density lipoprotein (HDL) cholesterol. The Immunolite Diagnostic Products Co, Los Angeles, CA, USA with solid-phase chemiluminescent enzyme immunoassay kit was used to measure fasting insulin levels. As already reported [38,39,40], the intra-assay coefficient of variation (CV) was <5.5%.

### 2.7. Adherence to MD

A qualified nutritionist during a face-to-face interview assessed adherence to MD using the 14-point Mediterranean Diet Adherence Screener (MEDAS) developed for the PREvencion con DIetaMEDiterranea (PREDIMED) study [41], as previously reported [42,43,44]. For each item, a score from 0 to 1 can be assigned; therefore, MEDAS-derived MD score was calculated as follows: lowest adherence (Score 0–5), average adherence (Score 6–9) and highest adherence (Score ≥ 10) [41].

### 2.8. Evaluation of Chronotype

Chronotype was obtained with the questionnaire MEQ [6], including 19 items about the subjective sensation of sleep and energy during the day and the preferred time to rest and to carry out demanding activities, with a score ranging from 16–84. Based on the score obtained, the participants were classified as being a morning (59–86), neither (42–58), or evening (16–41) chronotype, as previously reported [45]. 

### 2.9. Statistical Analysis

Data were collected and analyzed using the MedCalc^®^ package and SAS 9.3 program. The data distribution was evaluated by Kolmogorov-Smirnov test and the abnormal data were normalized by logarithm. Skewed variables (age, BMI, WC, SBP, DBP, circulating TMAO concentrations, triglycerides, total cholesterol, LDL cholesterol, HDL cholesterol, AST, ALT, γGT, MEDAS-derived MD score, and chronotype score) were converted into figures and tables. Results have been described as mean ± standard deviation (SD) or percentage/number. 

Differences according to sex and lifestyle habits were analyzed by Student’s unpaired *t*-test, while the differences among the classes of BMI and categories of MEDAS and chronotype were evaluated by ANOVA followed by the Bonferroni *post hoc* test. The chi square (χ^2^) test was used to evaluate the differences in the frequency distribution of physical activity, smoke, and MEDAS categories. The correlations between the study variables were performed using Pearson *r* correlation coefficients after adjusting for BMI and MEDAS-derived MD score. Proportional Odds Ratio (OR) models, 95% Interval Confidence (IC), and R^2^ were used to assess the associations among both circulating TMAO concentrations and chronotype scores on sex, lifestyle habits, and MEDAS categories. A linear regression was performed with circulating TMAO concentrations as the dependent variable and chronotype score, age, physical activity, smoking, BMI, and MEDAS-derived MD score as independent variables. Tolerance and variance inflation factor were calculated to determine the presence of collinearity. In addition, a logistic regression analysis was performed to assess the association between chronotype score and circulating TMAO concentrations above the median (6.80 µM). A restricted cubic spline analysis was performed to smooth the nonlinear relationship between chronotype score predictive of circulating TMAO concentrations and predict the factor levels that explain the pattern of linear correlation within variables. A separate curve was fit to each segment. The location of the knots was set according to the three categories of the chronotype: evening (16–41), neither (42–58), and morning (59–86) chronotype [6]. 

## 3. Results

The study population consisted of 247 participants, 147 females (49.5%) and 100 males (40.5%), aged 20–68 years. Ninety-seven subjects practiced physical activity at least 5 days a week (39.3%), while current smokers were 86 individuals (34.8%). BMIs ranged from 19 to 59 kg/m^2^. 

Table 1 summarizes the lifestyle information, BMI, WC, blood pressure, metabolic profile, nutritional parameter, and chronotype in the total study population. As shown in Table 1, most of the participants were normal weight (58.7%), had an average adherence to the MD (40.9%) and a morning chronotype type (62.3%).

Table 2 reports circulating TMAO concentrations and the chronotype score according to sex, smoking, physical activity, BMI categories, and MEDAS categories. Circulating TMAO concentrations and the chronotype score were significantly lower and higher, respectively, either in females (*p* < 0.001), or among physically active subjects or non-smokers (*p* < 0.001). Using the BMI and MEDAS categories to stratify the sample, circulating TMAO concentrations increased significantly along with the increase in BMI and decreased with increased MEDAS-derived MD score (*p* < 0.001). Contrariwise, the score of chronotype reduced significantly along with the increase in BMI and increased with a higher MEDAS-derived MD score (*p* < 0.001).

Table 3 reports sex, age, lifestyle information, BMI, WC, blood pressure, metabolic profile, and nutritional parameter in the study population grouped based on chronotype categories. Subjects with morning and evening chronotypes were older than those with the evening chronotype (*p* < 0.001). Participants with the morning chronotype followed a healthier lifestyle; in particular, they performed more regular physical activity (*p* = 0.006) and most of them were non-smokers (*p* = 0.004). Subjects with the morning chronotype had the lowest BMI (*p* < 0.001), WC (*p* < 0.001), PAS (*p* = 0.013), and the best metabolic profile compared to subjects with the other chronotypes. In addition, subjects with the morning chronotype had the highest MEDAS-derived MD score (*p* < 0.001), with the highest percentage of subjects with higher adherence to the MD (*p* < 0.001), as shown in Table 3.

**Figure 1:**
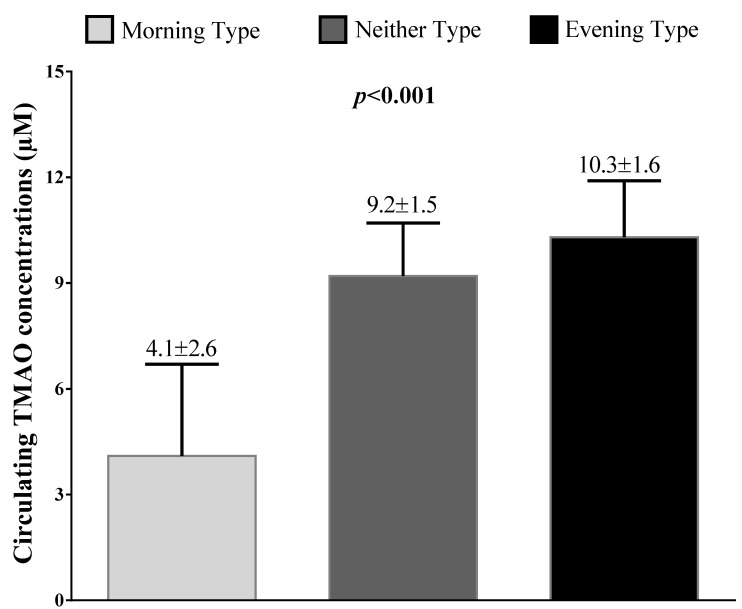
Circulating TMAO concentrations across chronotype categories. According to the three chronotype categories, circulating TMAO concentrations increased significantly, with the lowest values in the subjects with the morning chronotype category. Differences in the three groups were analyzed by ANOVA test, with the Bonferroni test as post hoc test. A *p* value in bold type denotes a significant difference (*p* < 0.05). TMAO, trimethylamine N-oxide.

Figure 1 reports circulating TMAO concentrations across the chronotype categories. In detail, stratifying the sample population according to the three chronotype categories, circulating TMAO concentrations increased significantly (*p* < 0.001), with the lowest score in the subjects with the morning chronotype category (4.1 ± 2.6 µM). 

### Correlation Analysis

The correlations between both circulating TMAO concentrations and chronotype scores with continuous variables age, BMI, WC, blood pressure, metabolic profile, and MEDAS-derived MD score are summarized in Table 4. All parameters evaluated in this study were significantly associated with both circulating TMAO concentrations and chronotype. 

**Figure 2:**
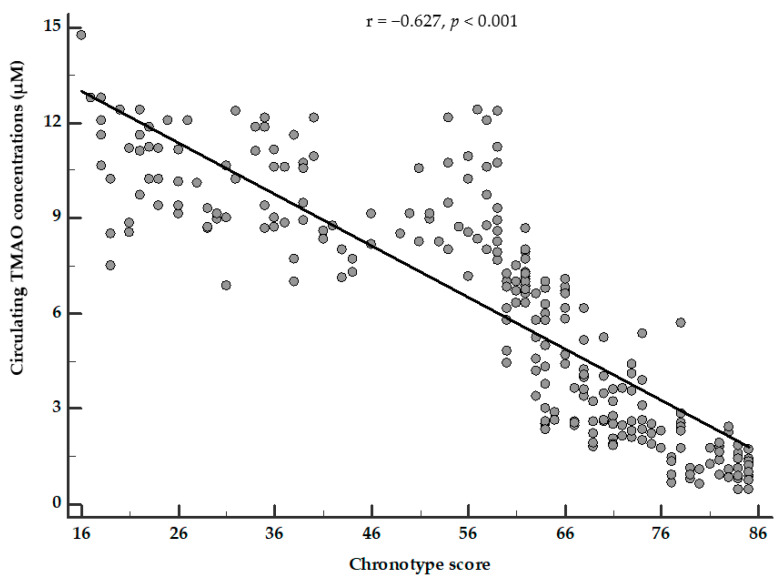
Partial correlation between circulating TMAO concentrations and chronotype score. Circulating TMAO concentrations were negatively associated with the chronotype score, after adjusting for BMI and MEDAS-derived MD score. Correlations between variables were performed using Pearson *r* correlation coefficients. A *p* value in bold type denotes a significant difference (*p* < 0.05). TMAO, trimethylamine N-oxide.

The correlation between circulating TMAO concentrations and the chronotype score was still evident (*p* < 0.001) after adjusting for BMI and the MEDAS-derived MD score (Figure 2). 

The results of a bivariate proportional OR model carried out to assess the association of both circulating TMAO concentrations and chronotype score with the categorical variables are reported in Table 5. Both circulating TMAO concentrations and the chronotype score were significantly associated with sex (*p* < 0.001), smoking (*p* < 0.001), and physical activity (*p* < 0.001). In addition, the highest circulating TMAO concentrations were associated with the lowest adherence to the MD (OR 1.53, *p* < 0.001) and the evening chronotype (OR 2.31, *p* < 0.001). Likewise, the lowest chronotype score was associated with the lowest adherence to the MD (OR 0.95, *p* < 0.001) (Table 5).

We performed a linear regression model to evaluate the association between circulating TMAO concentrations and the chronotype score, adjusting for potential confounders including age, BMI, waist circumference, physical activity, smoking, and MEDAS-derived MD score. In this model, the chronotype score was strongly associated with circulating TMAO concentrations (β = −0.479, *t* = −12.08, and *p* < 0.001). Results are reported in Table 6.

A logistic regression analysis was performed to assess the association between the chronotype score and circulating TMAO concentrations above the median (6.80 µM). In this model, the chronotype score was significantly associated with the circulating TMAO concentrations above the median value (OR = 0.50, *p* < 0.001, 95% IC = 0.38 to 0.65, R^2^ = 0.66).

**Figure 3:**
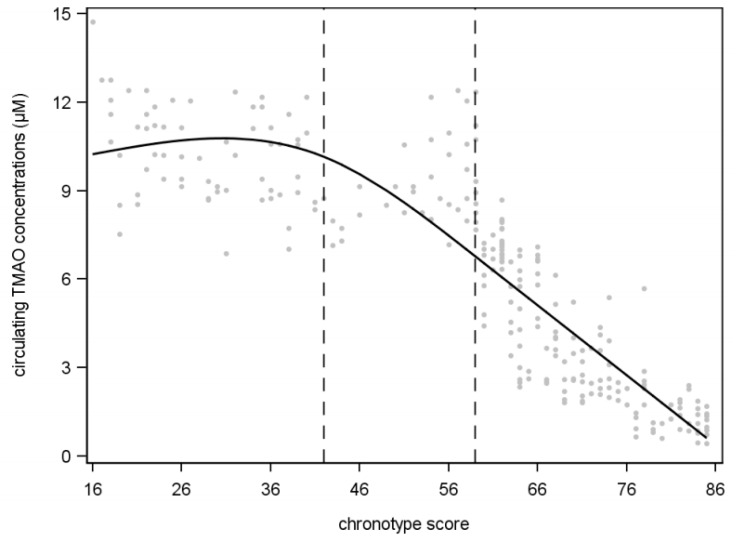
Association between circulating TMAO concentrations and chronotype score based on restricted cubic spline model in study population. A chronotype score ≥59 demonstrated a more significant inverse linear relationship with circulating TMAO concentrations compared with knots <59 (neither chronotype) and <41 (evening chronotype).

A restricted cubic spline analysis was then used to obtain the range of values of the chronotype score that split up with knots (evening, neither, and morning chronotype) defining the relationship with circulating TMAO concentrations at the end of one segment and the start of the next. In particular, a chronotype score ≥59 (*p* < 0.001, estimator = −0.0037, standard error = 0.0004, R^2^ = −0.824) demonstrated a more significant inverse linear relationship with circulating TMAO concentrations compared with knots <59 (neither chronotype) and <41 (evening chronotype); Figure 3. 

## 4. Discussion

In this cross-sectional observational study, we investigated circulating TMAO concentrations, a gut-derived metabolite that has proven to be a marker of gut dysbiosis, and cardiometabolic risk in three different chronotypes (morning, evening, and neither) according to sex, lifestyle habits, BMI, and adherence to the MD. The morning chronotype was observed in 62.3% of the subjects and was associated with the lowest anthropometric measurements, the better metabolic profile, and the higher adherence to the MD compared to intermediate and evening chronotypes. In addition, the highest percentage of morning chronotype was present in women, non-smokers, and physically active subjects. Stratifying the sample population according to the three chronotype categories, circulating TMAO concentrations decreased significantly along with the chronotype score, with the lowest values in the subjects with the morning chronotype category. As expected, while circulating TMAO concentrations were positively correlated with altered anthropometric measurements, blood pressure, metabolic profile, and lower MEDAS-derived MD score, the chronotype score was inversely correlated with those variables. To the best of our knowledge, this is the first study that reported a significant inverse correlation between circulating TMAO concentrations and the chronotype score, independently of common potential confounding covariates, such as anthropometric measurements and adherence to the MD. In particular, lower chronotype scores were mostly associated with higher circulating TMAO concentrations. Finally, using a restricted cubic spline analysis, we found that the morning chronotype demonstrated a more significant inverse linear relationship with circulating TMAO concentrations compared with neither and evening chronotypes. 

Chronotype can be defined as an attitude of behavioral preference for eveningness or morningness, or an individual measurement based on the timing of reported behaviors related to the sleep–wake cycle [46]. On the other side, in a given population, both genetic and environmental factors, including diet and gut microbiota, influence the distribution of chronotypes [2,46,47,48]. In particular, subjects with the evening chronotype are often chronically affected by misalignment in biological rhythms and irregular sleep and wake-up patterns, with a preference for nocturnal activities [49]; these conditions may increase, in the long term, the levels of both oxidative stress and chronic inflammation and alter the intestinal microbiota, thus increasing the risk of chronic diseases [50]. Consistently, a growing amount of evidence indicates that the evening chronotype is associated with chronic non-communicable diseases, such as cardiovascular diseases, obesity, metabolic syndrome [7,51,52], and gut dysbiosis [2,46,47,48]. Lately, the role of the circadian rhythm in the regulation of the gastrointestinal tract has been gaining a lot of interest [53]. Of interest, Nojkov et al. [54] reported that circadian alterations associated with shift work can lead to an increased incidence of diseases of the digestive system.

TMAO is a gut-derived metabolite depending on the function of the intestinal barrier, which is associated with an increased risk of metabolic syndrome, cardiovascular disease, and mortality [55]. Besides the gut microbiota, circulating TMAO concentrations are determined by many factors, such as age, gender, and dietary nutrients [15]. We previously reported that the circulating TMAO concentrations were negatively associated with a healthy dietary pattern, such as MD and 25-OH vitamin D levels [35,36]. In addition, we also found that the circulating TMAO concentrations represented an early biomarker of adipose dysfunction and non-alcoholic fatty liver disease [36]. In this context, the present study further increased the knowledge on the link between circulating TMAO concentrations and the adherence to the MD, as we evidenced that higher circulating TMAO concentrations were associated with an unhealthy lifestyle expressed not only by the low adherence to the MD, but also by the evening chronotype, a trait that best expresses the individual circadian preference in behavioral and biological rhythms and that has been previously associated with gut dysbiosis [2,10,46,47,48]. 

In line with our findings, it is well described that the low chronotype score, characterized by the evening chronotype on the one side, and higher circulating TMAO concentrations on the other side, is associated with an unhealthy metabolic profile, gut dysbiosis, and higher cardiovascular risk [7,56,57,58]. Thus, it is tempting to speculate on the association between the evening chronotype and gut dysbiosis, and the increased circulating TMAO concentrations, as the product of the oxygenation of TMA, might represent a biomarker of the increased intestinal translocation of TMA linked to gut dysbiosis and the altered intestinal barrier. 

**Figure 4:**
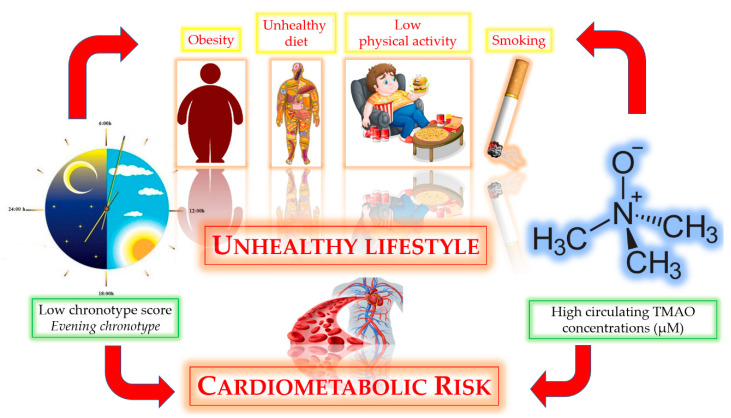
The possible bidirectional relationship between the evening chronotype and high circulating TMAO concentrations.

As a potential translational application, this study suggests that the association between evening chronotype and high circulating TMAO concentrations might potentiate the cardiometabolic risk. The possible bidirectional relationship between chronotype score and circulating TMAO concentrations has been resumed in Figure 4. 

This association might be of strategic utility considering that a dose–response meta-analysis of clinical studies indicated that the risk of all-cause mortality increased by 7.6% per 10 μM increment of circulating TMAO concentrations [59]. Nevertheless, it should be considered that the 19-item MEQ chronotype questionnaire is easy to administer and to analyze, while the detection of circulating TMAO concentrations requires venous sampling and dedicated laboratory facilities.

The main limitation of this study is that the cross-sectional design does not allow us to identify any causal associations between the variables analyzed. Therefore, we cannot establish the prognostic value of the chronotype score for predicting circulating TMAO concentrations. Secondly, we did not include in this study the metagenome analysis to explore gut microbiota dysbiosis in subjects with different chronotypes closely. However, it is well known that the very changeable composition of the gut microbiota might potentially hinder the robustness and reproducibility of microbiota—disease associative studies [20,60,61,62]. In addition, circulating TMAO concentrations have recently been included among the reliable biomarkers for gut dysbiosis [17]. A further limitation was that we did not include other metabolites involved in the same pathway of TMAO, such as carnitine, betaine, or choline. Nevertheless, the relationships between circulating TMAO concentrations and chronic non-communicable diseases were also independent of choline or other metabolites [63,64,65]. A further issue could arise from the use of the 14-item MEDAS questionnaire in an Italian sample population. The 14-item MEDAS questionnaire was previously validated for the Spanish population in the PREDIMED study. Nevertheless, the 14-item MEDAS questionnaire has been widely applied as a suitable tool for identifying the dietary habits of a population different from the Spanish one in several other countries around the world. In particular, very recently the 14-item MEDAS questionnaire was simultaneously validated by García-Conesa, M.T. et al. (2020) [66] in different Mediterranean countries, including Italy. The results of this study supported a general fair-to-moderate concordance for many of the food items of the 14-item MEDAS questionnaire principally in several Southern European countries around the Mediterranean area, thus pointing out the validity of this questionnaire as a rapid and flexible tool to evaluate the adherence to the MD. Finally, although the influence of chronotype upon sleep behavior is well recognized, we did not evaluate sleep quality in this study.

A major strength of this cross-sectional study is the strong characterization of our sample population, with the exclusion of conditions that can alter the metabolism of TMAO, such as type 2 diabetes and impaired kidney function. In addition, the single-center study allowed us to increase the homogeneity of the sample. Furthermore, we included a variety of potential covariates, such as adherence to the MD, anthropometric measurements and lifestyle, to limit the effect of confounding factors on the association of circulating TMAO concentrations with the chronotypes. A further strength was the assessment of circulating TMAO concentrations in plasma samples rather than in urine ones. Circulating TMAO concentrations are largely dependent upon dietary intake and urinary excretion, with an overlap in distribution among both healthy and diseased populations [67]. However, Krüger et al. [68] reported that although TMAO concentrations in plasma and urine might be differentially affected depending on the food source, the current fish consumption, one of the items included in the MEDAS-derived MD score, affected more urinary than plasma TMAO. In addition, the chronotype questionnaire was evaluated using a validated questionnaire, the 19-item MEQ questionnaire [6]. In particular, this questionnaire is poorly time demanding, requires poor collaboration from individuals, and provides a clear result to the interviewees immediately after finishing the questionnaire. Moreover, the MEQ questionnaire is not self-reported as it is face-to-face, administered by only one expert nutritionist to limit any bias regarding proper completion of the questionnaire and to avoid inter-operator variability.

## 5. Conclusions

In summary, the current study presented the first evidence that high circulating TMAO concentrations were associated with an unhealthy lifestyle expressed not only by low adherence to the MD, but also by the evening chronotype, a trait that best expresses the individual circadian preference in behavioral and biological rhythms. Our results support the evidence that that the evaluation of the chronotype should be included in the management of patients in a nutrition and dietetics clinic by a qualified nutritionist. This allows speculation on the plausible positive and cost-effective effects of carrying out dietary interventions according to the chronotype as support for clinical trials aiming to reduce circulating TMAO concentrations, and consequently, cardiometabolic risk. 

## Figures and Tables

**Table 1:** Lifestyle information, BMI, WC, blood pressure, metabolic profile, MEDAS-derived MD score, and chronotype of study participants.

Parameters	Mean ± SD or *n*. (%) *n*. 247
Lifestyle Habits	
Age (years)	36.6 ± 11.0
Smoking (yes)	86 (34.8%)
Physical activity (yes)	97 (39.3%)
Anthropometric measurement	
BMI (kg/m^2^)	28.8 ± 9.1
Normal weight	145 (58.7%)
Overweight	28 (11.3%)
Grade I obesity	21 (8.5%)
Grade II obesity	15 (6.1%)
Grade III obesity	38 (15.4%)
WC (cm)	98.6 ± 23.1
WC (cm) males	109.7 ± 22.3
WC (cm) females	91.1 ± 20.5
Blood pressure	
SBP (mmHg)	128.4 ± 12.8
DBP (mmHg)	80.6 ± 9.1
Metabolic profile	
Circulating TMAO concentrations (µM)	6.2 ± 3.6
Fasting glucose (mg/dL)	91.3 ± 14.5
Triglycerides (mg/dL)	117.5 ± 42.6
Total cholesterol (mg/dL)	160.6 ± 36.1
LDL cholesterol (mg/dL)	85.7 ± 40.6
HDL cholesterol (mg/dL)	51.4 ± 11.3
AST (U/L)	27.4 ± 13.9
ALT (U/L)	26.9 ± 14.0
γGT (U/L)	30.2 ± 16.5
Nutritional parameter	
MEDAS-derived MD score	8.1 ± 2.7
Low adherence to the MD	56 (22.7%)
Average adherence to the MD	101 (40.9%)
High adherence to the MD	90 (36.4%)
Chronotype	
Chronotype score	57.5 ± 19.3
Morning Type	154 (62.3%)
Neither Type	30 (12.2%)
Evening Type	63 (25.5%)

Most of the participants were normal weight (58.7%), had an average adherence to the MD (40.9%), and had a morning chronotype type (62.3%). Results were expressed as mean ± SD or number (%). BMI, body mass index; WC, waist circumference; SBP, systolic blood pressure; DBP, diastolic blood pressure; TMAO, trimethylamine N-oxide; LDL, low-density lipoprotein; HDL, high-density lipoprotein; AST, aspartate aminotransferase; ALT, alanine aminotransferase; γGT, gamma-glutamyl transferase; MEDAS, Mediterranean Diet Adherence Score; MD, Mediterranean diet.

**Table 2:** Circulating TMAO concentrations and chronotype score in the study population according to sex, lifestyle habits, and BMI/MEDAS categories.

Parameters		Circulating TMAO Concentrations (µM)	*p*-Value	Chronotype Score	*p*-Value
Sex	Males (*n* 100, 40.5%)	8.4 ± 3.1	**<0.001**	46.7 ± 19.2	**<0.001**
	Females (*n* 147, 49.5%)	4.8 ± 3.3		64.8 ± 15.7	
Smoking	Yes (*n* 86, 34.8%)	7.6 ± 3.4	**<0.001**	49.9 ± 19.8	**<0.001**
	No (*n* 161, 65.2%)	5.5 ± 3.6		61.5 ± 17.9	
Physical activity	Yes (*n* 97, 39.3%)	4.7 ± 3.2	**<0.001**	63.9 ± 16.8	**<0.001**
	No (*n* 150, 60.7%)	7.3 ± 3.7		53.3 ± 19.7	
BMI	Normal weight (*n* 145, 58.7%)	3.7 ± 2.3	**<0.001**	69.8 ± 10.4	**<0.001**
	Overweight (*n* 28, 11.3%)	8.2 ± 0.7		48.4 ± 14.4	
	Grade I obesity (*n* 21, 8.5%)	9.0 ± 0.9		39.7 ± 12.7	
	Grade II obesity (*n* 15, 6.1%)	9.9 ± 0.8		40.6 ± 14.0	
	Grade III obesity (*n* 38, 15.4%)	11.5 ± 0.9		33.5 ± 14.3	
MEDAS-derived MD score	Low adherence to the MD (*n* 56, 22.7%)	9.5 ± 2.9	**<0.001**	41.9 ± 18.7	**<0.001**
	Average adherence to the MD (*n* 101, 40.9%)	6.7 ± 3.1		56.4 ± 17.6	
	High adherence to the MD (*n* 90, 36.4%)	3.8 ± 2.7		68.4 ± 13.8	

Both circulating TMAO concentrations and the chronotype score were significantly different in all categories examined (*p* < 0.001). Differences according to sex and lifestyle habits were analyzed by Student’s unpaired *t*-test, while the differences among the classes of BMI and MEDAS were evaluated by ANOVA followed by the Bonferroni post hoc test. Results were expressed as mean ± SD. A *p* value in bold type denotes a significant difference (*p* < 0.05). TMAO, trimethylamine N-oxide; BMI, body mass index; MEDAS, Mediterranean Diet Adherence Score; MD, Mediterranean diet.

**Table 3:** Sex, age, lifestyle habits, anthropometric measurements, blood pressure, metabolic profile, and nutritional parameter in the study population grouped based on chronotype categories.

Parameters	Morning Type *n* = 154, 62.3%	Neither Type *n* = 30, 12.2%	Evening Type *n* = 63, 25.5%	*p*-Value
Gender				
Males (*n*, %)	41, 26.6%	12, 40.0%	47, 74.6%	χ^2^ = 42.7 ***p*** **< 0.001**
Females (*n*, %)	113, 73.4%	18, 60.0%	16, 25.4%	
Age (years)	38.5 ± 11.0	40.0 ± 11.5	30.3 ± 8.2	**<0.001**
Smoking				
Yes	42, 27.3%	12, 40.0%	32, 49.2%	χ^2^ = 11.3 ***p*** **= 0.004**
No	112, 72.7%	18, 60.0%	31, 50.8%	
Physical activity				
Yes	72, 46.8%	10, 33.3%	15, 23.8%	χ^2^ = 10.4 ***p*** **= 0.006**
No	82, 53.2%	20, 66.7%	48, 76.2%	
Anthropometric measurements				
BMI (kg/m^2^)	23.8 ± 4.0	32.9 ± 7.5	39.1 ± 9.3	**<0.001**
WC (cm)	87.4 ± 14.4	109.0 ± 21.3	121.1 ± 22.5	**<0.001**
Blood pressure				
SBP (mmHg)	120.6 ± 10.9	127.3 ± 12.5	130.9 ± 12.6	**0.013**
DBP (mmHg)	78.8 ± 7.9	80.9 ± 9.1	80.9 ± 9.5	0.692
Metabolic profile				
Fasting glucose (mg/dL)	86.1 ± 12.4	96.5 ± 13.3	101.4 ± 13.8	**<0.001**
Triglycerides (mg/dL)	111.4 ± 41.1	124.7 ± 33.0	129.2 ± 47.4	**0.012**
Total cholesterol (mg/dL)	151.7 ± 30.5	184.7 ± 29.8	170.5 ± 43.4	**<0.001**
LDL cholesterol (mg/dL)	75.6 ± 35.3	113.9 ± 33.9	96.8 ± 46.7	**<0.001**
HDL cholesterol (mg/dL)	53.8 ± 11.2	45.9 ± 9.9	47.5 ± 10.3	**<0.001**
AST (U/L)	24.4 ± 10.8	33.3 ± 17.5	30.2 ± 17.3	**0.001**
ALT (U/L)	24.0 ± 13.2	34.9 ± 16.2	31.9 ± 12.1	**<0.001**
γGT (U/L)	26.1 ± 10.5	35.1 ± 19.6	37.7 ± 22.9	**<0.001**
Nutritional parameter				
MEDAS-derived MD score	9.0 ± 2.4	7.1 ± 2.5	6.2 ± 2.4	**<0.001**
Low adherence to the MD	15, 9.7%	9, 30.0%	32, 50.8%	χ^2^ = 44.0, ***p* < 0.001**
Average adherence to the MD	61, 39.6%	16, 53.3%	24, 38.1%	χ^2^ = 2.2, *p* = 0.328
High adherence to the MD	78, 50.6%	5, 16.7%	7, 11.1%	χ^2^= 35.9, ***p* < 0.001**

Participants with the morning chronotype followed a healthier lifestyle, had the lowest anthropometric measurements, SBP, and the best metabolic profile compared to the other chronotypes. Results are expressed as mean ± SD. Differences in the three groups were analyzed by ANOVA test, with the Bonferroni test as post hoc test. A *p* value in bold type denotes a significant difference (*p* < 0.05). BMI, body mass index; WC, waist circumference; SBP, systolic blood pressure; DBP, diastolic blood pressure; TMAO, trimethylamine N-oxide; LDL, low-density lipoprotein; HDL, high-density lipoprotein; AST, aspartate aminotransferase; ALT, alanine aminotransferase; γGT, gamma-glutamyl transferase; MEDAS, Mediterranean Diet Adherence Score; MD, Mediterranean diet.

**Table 4:** Correlations between circulating TMAO concentrations with chronotype scores, age, BMI, WC, blood pressure, metabolic profile, and MEDAS-derived MD score.

Parameters	Circulating TMAO Concentrations (µM)		Chronotype Score	
	r	*p*-Value	r	*p*-Value
Age (years)	−0.277	**<0.001**	0.361	**<0.001**
Anthropometric measurements				
BMI (kg/m^2^)	0.842	**<0.001**	−0.746	**<0.001**
WC (cm)	0.785	**<0.001**	−0.670	**<0.001**
Blood pressure				
SBP (mmHg)	0.357	**<0.001**	−0.286	**0.003**
DBP (mmHg)	0.245	**0.013**	−0.042	0.672
Metabolic profile				
Fasting glucose (mg/dL)	0.519	**<0.001**	−0.439	**<0.001**
Triglycerides (mg/dL)	0.253	**<0.001**	−0.175	**0.006**
Total cholesterol (mg/dL)	0.264	**<0.001**	−0.133	**0.037**
LDL cholesterol (mg/dL)	0.255	**<0.001**	−0.121	**0.050**
HDL cholesterol (mg/dL)	−0.261	**<0.001**	0.140	**0.028**
AST (U/L)	0.261	**<0.001**	−0.194	**0.002**
ALT (U/L)	0.195	**0.002**	−0.137	**0.031**
γGT (U/L)	0.320	**<0.001**	−0.297	**<0.001**
Nutritional parameter				
MEDAS-derived MD score	−0.632	**<0.001**	0.525	**<0.001**

All parameters evaluated in this study were significantly associated with both circulating TMAO concentrations and the chronotype score. Correlations between variables were performed using Pearson *r* correlation coefficients. A *p* value in bold type denotes a significant difference (*p* < 0.05). BMI, body mass index; WC, waist circumference; SBP, systolic blood pressure; DBP, diastolic blood pressure; TMAO, trimethylamine N-oxide; LDL, low-density lipoprotein; HDL, high-density lipoprotein; AST, aspartate aminotransferase; ALT, alanine aminotransferase; γGT, gamma-glutamyl transferase; MEDAS, Mediterranean Diet Adherence Score; MD, Mediterranean diet.

**Table 5:** Bivariate proportional odds ratio model to assess the association between both circulating TMAO concentrations and chronotype score with sex, lifestyle habits, MEDAS/chronotype categories.

Parameters	Circulating TMAO Concentrations (µM)				Chronotype Score			
	OR	*p*-Value	95% IC	R^2^	OR	*p*-Value	95% IC	R^2^
Sex	1.37	**<0.001**	1.25–1.50	0.22	0.95	**<0.001**	0.93–0.96	0.20
Smoking	1.18	**<0.001**	1.09–1.28	0.07	0.97	**<0.001**	0.96–0.98	0.08
Physical activity	0.08	**<0.001**	0.75–0.87	0.12	1.03	**<0.001**	1.02–1.05	0.07
MEDAS categories								
Low adherence to the MD	1.53	**<0.001**	1.34–1.74	0.23	0.95	**<0.001**	0.93–0.96	0.17
Average adherence to the MD	1.06	**0.007**	0.98–1.13	0.01	0.69	**0.004**	0.98–1.00	0.01
High adherence to the MD	0.69	**<0.001**	0.63–0.76	0.26	1.07	**<0.001**	1.04–1.09	0.19
Chronotype								
Morning Type	0.27	**<0.001**	0.18–0.40	0.59	-	-	-	-
Neither Type	1.34	**<0.001**	1.17–1.53	0.09	-	-	-	-
Evening Type	2.31	**<0.001**	1.82–2.92	0.43	-	-	-	-

Both circulating TMAO concentrations and chronotype score were significantly associated with sex, smoking, and physical activity. In particular, the highest circulating TMAO concentrations were associated with the lowest adherence to the MD and the evening chronotype. Likewise, the lowest chronotype score was associated with the lowest adherence to the MD. Bivariate proportional OR model, 95% IC, and R^2^. A *p* value in bold type denotes a significant difference (*p* < 0.05). TMAO, trimethylamine N-oxide; MEDAS, Mediterranean Diet Adherence Score; MD, Mediterranean diet; OR, odds ratio; IC, Interval Confidence.

**Table 6:** Linear regression model for the association between circulating TMAO concentrations and chronotype score, adjusting for potential confounders including age, physical activity, smoking, BMI, MEDAS-derived MD score.

Parameters	Linear Regression Model							
	Non-Standardized Coefficients		Standardized Coefficients				Collinearity Statistics	
	T	SE	β	*t*	*p*-Value	95% IC	Tolerance	VIF
Chronotype score	−0.090	0.007	−0.479	−12.08	**<0.001**	−0.11 to −0.08	0.393	2.54
MEDAS-derived MD score	−0.192	0.043	−0.141	−4.50	**<0.001**	−0.28 to −0.11	0.627	1.60
BMI (kg/m^2^)	0.098	0.025	0.247	3.86	**<0.001**	0.05 to 0.15	0.151	6.60
Waist circumference (cm)	0.025	0.009	0.156	2.75	**0.006**	0.01 to 0.04	0.194	5.16
Smoking	0.457	0.202	0.060	2.26	**0.025**	0.06 to 0.86	0.883	1.13
Physical activity	−0.399	0.198	−0.053	−2.01	0.045	−0.79 to 0.01	0.877	1.14
Age (years)	0.015	0.009	0.045	1.68	0.094	−0.01 to 0.03	0.849	1.18

Using a linear regression model, the chronotype score was strongly associated with circulating TMAO concentrations. TMAO, trimethylamine N-oxide; BMI, body mass index; MEDAS, Mediterranean Diet Adherence Score; MD, Mediterranean diet; SE, standard error; IC, Interval Confidence; VIF, variance inflation factor.

## Data Availability

Results attained in this study are included in the manuscript. Individual data are not publicly available due to ethical restrictions.
